# Preoperative Protein Profiling Among Postoperative Cognitive Dysfunction (POCD) Patients Following Open-Heart Surgery: A Systematic Review and Integrated Bioinformatic Analysis

**DOI:** 10.3390/ijms252212238

**Published:** 2024-11-14

**Authors:** Marjanu Hikmah Elias, Nazefah Abdul Hamid, Sofwatul Mokhtarah Maluin, Shamsir Aris, Suhaini Kadiman, Kamilah Muhammad Hafidz, Norsham Juliana

**Affiliations:** 1 Faculty of Medicine and Health Sciences, Universiti Sains Islam Malaysia, Bandar Baru Nilai 71800, Negeri Sembilan, Malaysia; marjanuhikmah@usim.edu.my (M.H.E.); nazefah@usim.edu.my (N.A.H.); sofwatulmal@usim.edu.my (S.M.M.); shamsir@usim.edu.my (S.A.); 2 Department of Anaesthesia and Intensive Care, Institut Jantung Negara, Kuala Lumpur 50400, Malaysia; suhaini@ijn.com.my (S.K.); dr.kamilah@ijn.com.my (K.M.H.)

**Keywords:** postoperative cognitive complications (POCD), cardiac surgery, biomarkers, protein expression profiling, cognitive assessment

## Abstract

The inability to accurately predict the occurrence of postoperative cognitive dysfunction (POCD) among open-heart surgery patients leads to concerning increases in POCD cases. Preoperative circulating biomarkers are important to identify as they are non-invasive and could provide an early prediction of POCD development, allowing for earlier and more strategized interventions. However, to date, no robust circulating biomarkers have proven effective for preoperative POCD prediction. This systematic review aims to synthesize current evidence on preoperative protein profiling among POCD patients following open-heart surgery. Thus, a thorough literature search employing PubMed, EBSCOhost, Scopus, and Science Direct was carried out. This combination of keywords was used as part of the search strategy: (“Postoperative cognitive decline” OR “Postoperative cognitive disorders” OR “Postoperative cognitive dysfunction” OR “Postoperative cognitive complications”) AND (“Thoracic Surgery” OR “Cardiac Surgery” OR “Heart Surgery”) AND (“Protein expression” OR proteomic OR “Protein profiling”). Eight hundred and twenty-nine studies were retrieved and only clinical studies reporting the circulating preoperative differentially expressed Proteins (DEPs) in the POCD patients were selected. Six studies were selected following the inclusion and exclusion criteria. Only one preoperative DEP and four immediate postoperative DEPs were extracted from the studies. All four proteins were selected for analysis using DAVID, STRING, and Cytoscape software. Due to the very low number of proteins, no clusters have been identified. This systematic review demonstrates the lack of POCD preoperative biomarkers for open-heart-surgery patients. Thus, it is suggested that more studies can be conducted to fill this gap.

## 1. Introduction

Postoperative cognitive dysfunction (POCD) is an undesired complication following open-heart surgery that affects a notable number of patients and leads to a decreased quality of life, increased healthcare costs, and potential long-term cognitive impairment. The updated definition of POCD is ‘delayed neurocognitive recovery’ that present within 30 days of surgery, or ‘postoperative neurocognitive disorder’ if present within 1 year after surgery [1]. POCD is more prevalent in patients who have undergone cardiac surgery compared to those who have had other types of surgery [2]. The prevalence of patients being classified with POCD after heart surgery is approximately 20% to 40%. It is a major concern as studies found that the patients continue to show signs of cognitive problems months or even years later [2,3]. These figures forecast the subsequent burden on healthcare systems and the economy as patients with POCD often require longer hospital stays, more rehabilitation services, and sometimes long-term care [2,4].

Recent studies have identified several important protein markers associated with POCD risk in cardiac surgery patients. Inflammatory mediators, such as C-reactive protein and various interleukins, have been found to have significant roles in POCD development. Aksoy et al. (2020) reported that patients who subsequently developed POCD after coronary artery bypass grafting are those with elevated preoperative levels of IL-6 and TNF-α [5]. Additionally, neurodegenerative markers, including tau protein and amyloid-β, were potential predictors of POCD. Higher preoperative concentrations of these proteins may indicate a predisposing risk of neurodegenerative processes that result in the higher susceptibility of the patients towards postoperative cognitive decline [6]. Oxidative stress markers have also emerged as potential predictors. Recent studies are advancing towards utilizing machine learning approaches in combination with plasma proteomics to develop predictive models with high accuracy in predicting cognitive decline. However, most studies focus on neurodegenerative decline in general without specific indications due to postoperative complications [7,8]. The integration of proteomic data with bioinformatic analysis has revealed enriched pathways and biological processes associated with POCD. These include complement and coagulation cascades, lipid metabolism pathways, and inflammatory signaling networks. These findings suggest that pre-existing dysregulation in these systems may contribute to the pathogenesis of POCD [9,10,11].

Given the substantial clinical and socioeconomic implications of POCD on patients, healthcare systems, and society at large, there is an urgency to develop novel predictive models and interventional strategies for mitigating cognitive decline following cardiac surgery. Preoperative protein profiling may serve as a promising avenue of investigation in this context. This approach involves the comprehensive analysis of protein expression patterns in patient serum or plasma samples prior to surgical intervention [12]. The distinct proteomic signatures associated with an increased POCD risk will allow the effective stratification of patients, hence implementing tailored perioperative management protocols. Such personalized approaches could potentially encompass modifications to surgical techniques, anesthetic regimens, and postoperative care pathways, with the ultimate goal of preserving cognitive function in high-risk individuals [13]. The integration of proteomic data with other clinical and demographic factors may yield robust predictive algorithms, facilitating more informed decision making and targeted preventive strategies in the management of cardiac surgery patients [14]. This systematic review aims to synthesize current evidence on preoperative protein profiling among POCD patients following open-heart surgery and integrate these findings through bioinformatic analysis.

## 2. Results

The search from the four databases, using the selected keywords, resulted in 829 possibly relevant studies. Based on the titles, 148 duplicates were removed and the abstracts of the remaining 681 studies were reviewed. After the abstract was evaluated, 664 studies were eliminated. Then, all the full text from the remaining 17 papers were retrieved. Eleven original articles that did not suit the inclusion and exclusion criteria were eliminated following a comprehensive reading and analysis of the full text. Finally, six studies were selected for the systematic review based on the inclusion and exclusion criteria. These six studies were published between 2016 and 2022. The total number of participants included in this systematic review is 461. The sample size of each study ranged from 32 to 178. All studies used peripheral blood as the sample collected for the protein analysis. The summary of the features of the included studies is presented in Table 1. Additionally, Table 2 shows the baseline characteristics of the POCD and non-POCD groups, along with the statistical significance of the differences observed between them.

### 2.1. Patient Recruitment and Sample Collection Details

Four hundred sixty-one patients from six studies were included in this systematic review. The included patients were aged between 20 and 90 years old. All included studies collected peripheral blood for protein expression analysis using ELISA or the immunoturbidimetric method. However, the demographic profile of the patients was not further described in any of the studies. Only Baktiar et al. (2020) [18] did not mention the anesthesia used during the surgery. Various sets of tests were used to identify POCD.

### 2.2. Study Quality

Table S1 contains a comprehensive quality assessment of the included studies. In this systematic review, one study with a moderate risk of bias and five high-quality studies with a low risk of bias were included.

### 2.3. DEPs in POCD Patients

Only one protein was reported to be significantly upregulated in POCD patients before the cardiac surgery, which is the CRP. The other 10 proteins were not significantly different between POCD and non-POCD patients before the surgery. Four proteins, CRP, TREM2, RTN1, and S100B, were upregulated significantly post-surgery (within 24 h after the surgery) in POCD patients. However, post-surgery, six tested proteins were not differentially regulated in POCD patients. Table 3 summarizes the reported DEPs in the patients identified as having POCD in each study.

### 2.4. Protein–Protein Interaction Network

**Figure 1:**
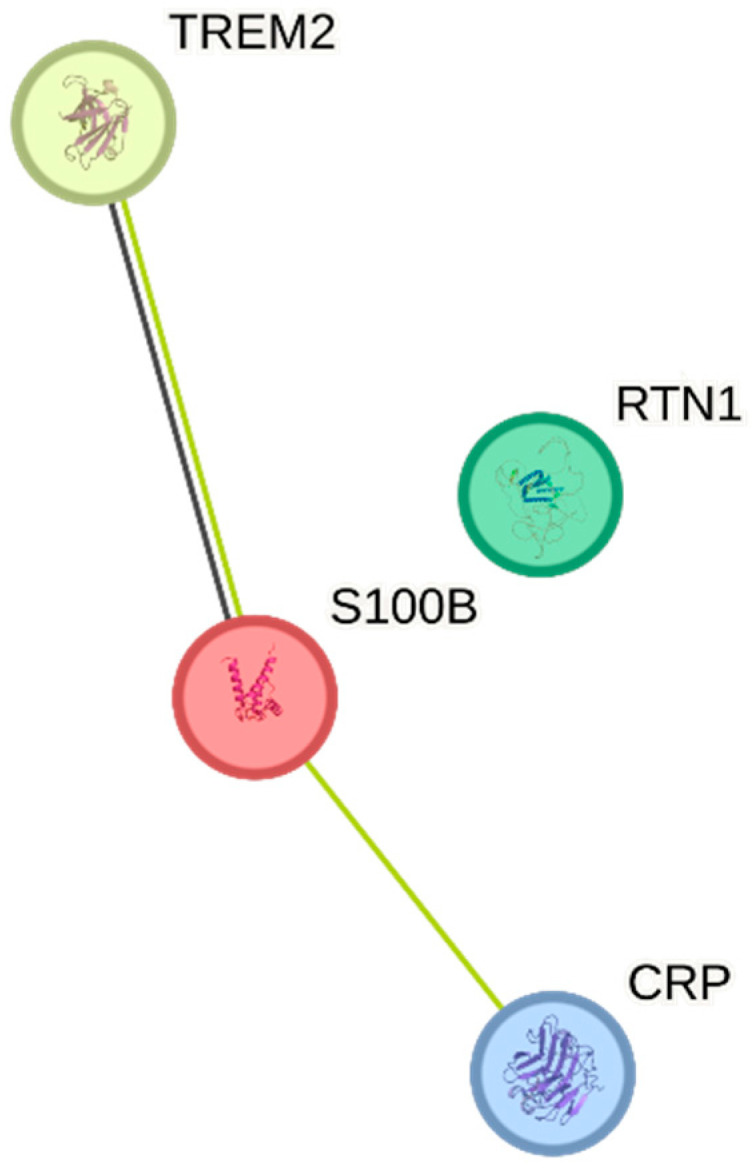
PPI network of the DEPs collected from the selected studies.

Biological pathways of the proteins retrieved from the selected studies were identified using the Protein–Protein Interaction (PPI) Network and Modular Analysis. A PPI network comprising four nodes and two edges (an average local clustering coefficient of 0.5; PPI enrichment *p*-value of 0.011) was constructed by filtering four significantly differentiated proteins from the selected studies. The network’s data were sent from STRING to Cytoscape for gene clustering. No cluster was found from the PPI network complex following the Molecular Complex Detection (MCODE) analysis. Figure 1 shows the PPI network derived from the DEPs derived from all the selected studies.

### 2.5. Functional Enrichment of the Network

One gene ontology (GO) on the molecular function was found to be related to the PPI network of POCD development among patients who underwent open-heart surgery. The GO-term is GO:0030169, contributing to low-density lipoprotein particle binding. The disease–gene association suggests that the genes reported in the selected studies are related to cardiac arrest (DOID:0060319). However, there was no significant pathway enrichment observed in the Biological Process (Gene Ontology), Cellular Component (Gene Ontology), and KEGG Pathways.

## 3. Discussion

### 3.1. POCD Assessment Method

POCD is a significant complication that often follows cardiac surgery, especially among older adults. Its diagnosis relies on standardized neuropsychological assessments administered both pre- and post-surgery. These assessments systematically evaluate key cognitive domains including memory, attention, language, orientation, and executive function [21]. Table 4 summarizes the cognitive assessment tools used in studies for identifying POCD.

In the reviewed studies, the most frequently employed cognitive assessment tools are the MMSE, MoCA, SDMT, TMT, and RAVLT, chosen for their reliability, ease of use, and ability to detect various cognitive impairments associated with POCD. Some research incorporated up to eight different cognitive assessments to increase the accuracy of POCD detection [15,20]. A POCD diagnosis was typically defined by a decline of more than 20% from the preoperative performance in two or more postoperative tests, a criterion also used by Silva et al. 2016 [20], Baktiar et al. 2020 [18], and Szwed et al. 2020 [17].

Another important factor in selecting a cognitive assessment tool is the sensitivity and specificity of each method. The MMSE demonstrates a sensitivity of 85% to 92% and a specificity of 85% to 93% when using cutoff scores of 23 or 24 [22]. Conversely, the MoCA is notably sensitive in detecting mild cognitive impairment (MCI), with a sensitivity rate of 90%, significantly higher than the MMSE’s 18% [23]. This makes MoCA superior to the MMSE for detecting mild cognitive impairment, as shown in studies like Nurcahyo et al. (2021) [16], which used an adapted Indonesian version (MoCA-INA).

Additionally, the Symbol Digit Modalities Test (SDMT) demonstrates approximately 81% sensitivity and 78% specificity, making it a valuable tool for evaluating cognitive processing deficits [24]. This reliability underpins its widespread use in POCD studies. The Trail Making Test (TMT) and Rey Auditory Verbal Learning Test (RAVLT) are also frequently utilized for their effectiveness in assessing executive function and verbal memory, respectively, contributing to a comprehensive evaluation toolkit in POCD research.

Furthermore, it is important to note that POCD is defined as cognitive impairment occurring within 30 days post-surgery. Consequently, most studies conduct cognitive assessments within this timeframe. However, some studies, such as those by Wang et al. (2022) [15] and Silva et al. (2016) [20], extended their assessments to up to six months postoperatively. This extended duration allows for the evaluation of longer-term cognitive effects and the persistence of POCD. To facilitate data collection and reduce the loss-to-follow-up rate, these studies utilized the Telephone Interview for Cognitive Status (TICS). Notably, Wang et al. (2022) [15] found that POCD persisted for up to six months in patients who had undergone thoracoabdominal aortic dissection surgery.

Nevertheless, the chosen POCD assessment method must prioritize simplicity to avoid overburdening patients recovering from high-risk procedures. A recent study assessed the feasibility of MMSE as the sole tool for POCD evaluation and found that while the TMT, Digit Span, and Digit Symbol Substitution Test (DSST) showed moderate correlations with the MMSE, the Clock Drawing Test (CDT) had a weak correlation. Despite this, multiple linear regression analyses highlighted TMT Part B, Digit Span, DSST, and CDT as statistically significant and closely related to the MMSE [25]. This indicates that while the MMSE is effective on its own, incorporating additional tools such as TMT and DSST can provide a more comprehensive assessment without significantly complicating the process for patients. Collectively, these assessments offer valuable tools for examining post-surgical cognitive changes and inform targeted interventions to reduce the risks of POCD.

The outcomes of these cognitive assessments may be influenced by the types of anesthesia administered to the subjects. Five out of six studies reported the types of anesthesia used, with Wang et al. (2022) [15], Szwed et al. (2020) [17], and Silva et al. (2016) [20] utilizing propofol, an agent previously linked to a reduced incidence of postoperative cognitive dysfunction (POCD). Fentanyl or sufentanil were uniformly used in five studies, except for Baktiar et al. (2020) [18], which did not specify the types of anesthesia. These agents are associated with a greater potential to lower the incidence of POCD. However, sevoflurane, employed in studies by Wang et al. (2022) [15], Szwed et al. (2020) [17], and Nurcahyo et al. (2021) [16], has been reported as one of the least effective sedative agents in reducing the POCD incidence.

To date, the most prominent anesthetic reported to significantly reduce the incidence of POCD is dexmedetomidine (Dex) [25,26]. However, none of the studies included in this review reported the use of Dex. Given the potential of the anesthetic choice to influence POCD outcomes, future studies and reviews investigating differences in biomarker levels should consider including the anesthetic type as a confounding factor.

**Table 4:** Overview of Cognitive Assessment Methods for Postoperative Cognitive Dysfunction (POCD).

Assessment Method	Description	Key Cognitive Domains Assessed	References
Mini Mental State Examination (MMSE)	Includes questions assessing orientation to both time and place, along with tasks that involve repeating a phrase, recalling a sequence of words, following verbal and written commands, and performing basic calculations.	Arithmetic, Memory, Orientation, Language	[22]
Montreal Cognitive Assessment (MoCA)	The assessment consists of various tasks such as delayed recall of a list of words, serial subtraction, visuospatial abilities, and abstraction.	Memory, Attention, Language, Executive Function	[23]
Symbol Digit Modalities Test (SDMT)	The test requires individuals to match specific symbols with corresponding numbers within a set time frame, thereby evaluating key cognitive abilities crucial for information processing.	Attention, Visual Scanning, Motor Speed	[27]
Digit Span Tests (DSFT and DSBT)	The test includes the Digit Span Forward Test (DSFT) and the Digit Span Backward Test (DSBT), by requiring participants to repeat a sequence of numbers both forward and backward.	Working Memory	[28]
Rey Auditory Verbal Learning Test (RAVLT)	Measuring the ability to recall a list of words both immediately and after a delay. Immediate and delayed recall of word lists.	Verbal Memory, Learning	[29]
Stroop Color–Word Test (SCWT)	Measures cognitive flexibility and inhibitory control by naming ink colors of color names.	Executive Function, Inhibitory Control	[30]
Confusion Assessment Method—Intensive Care Unit (CAM-ICU)	Specifically designed for ICU patients to assess acute confusion and changes in mental status.	Acute Confusion, Mental Status	[31]
Trail Making Test (TMT)	TMT-A assesses processing speed and visual attention, while TMT-B evaluates executive function and cognitive flexibility, inhibition control, and working memory.	Processing Speed, Visual Attention, Executive Function	[32]
Telephone Interview Cognitive Status (TICS)	Alternative for remote cognitive assessment via phone; useful for follow-up studies.	Memory, Orientation, Attention, Language, Arithmetic	[33]

### 3.2. Biomarkers for POCD

Identifying preoperative and postoperative biomarkers for POCD is crucial to allow effective preventive measures for patients undergoing open-heart surgery. This systematic review reveals a lack of studies carried out on the preoperative and postoperative gene expression in POCD patients following open-heart surgery. Only six studies reported on the preoperative and postoperative protein expression. However, all the studies only targeted a few proteins. Only a single protein was studied by Nurcahyo et al. 2021 [16], Baktiar et al. 2020 [18], and He et al. (2017) [19] by utilizing a turbidimetric immunoassay or ELISA. Four proteins were studied by Szwed et al. 2020 [17] and Wang et al. 2022 [15], respectively.

POCD is a multifactorial disease that involves various pathways [14]. Thus, a single plex protein expression assay like ELISA is not robust enough to identify the best set of biomarkers for predicting the development of POCD among patients undergoing open-heart surgery. Instead, a high-throughput protein expression method such as whole-protein sequencing should be used to screen for more candidate proteins, and hence producing a more accurate prediction of POCD development. Thus, the preliminary results of the protein expression landscape and pathways involved in POCD development among open-heart surgery patients are crucial to be explored. From this systematic review, a few genes and pathways were found to be involved in POCD development and can be utilized as the preoperative and postoperative biomarkers for open-heart surgery patients.

In a study, the C-reactive protein (CRP) was reported to be upregulated among POCD patients during postoperative as well as during preoperative open-heart surgery [16]. The CRP is produced by the liver and released into the blood in response to tissue injury, inflammation, and infection. Thus, elevated CRP levels in the blood during the preoperative period among patients who develop POCD can be indicative of several underlying processes that may contribute to cognitive decline after surgery.

The CRP is a marker of systemic inflammation, and its elevation suggests an ongoing inflammatory response, leading to an increased risk of neuroinflammation, endothelial dysfunction, oxidative stress, and neuronal damage. Systemic inflammation can disrupt the integrity of the blood–brain barrier (BBB), allowing inflammatory mediators to enter the central nervous system (CNS) [34]. Once inflammatory mediators cross the BBB, they can activate microglia (the brain’s resident immune cells), leading to neuroinflammation. Neuroinflammation is known to contribute to cognitive impairment and neurodegenerative processes [35].

Patients with chronic inflammatory conditions such as cardiovascular diseases, diabetes, or autoimmune disorders often have elevated CRP levels [36]. Chronic inflammation makes the brain more vulnerable to additional stress. Subsequently, it predisposes these patients to higher risks of cognitive decline, especially when they undergo major surgical procedures that further stress the body.

Elevated CRP is also associated with endothelial dysfunction, which can impair the cerebral blood flow and oxygen delivery to the brain [37]. Poor vascular health can exacerbate the impact of surgical stress on the brain, leading to cognitive impairment. Patients with high preoperative CRP levels may have neurons that are already stressed or damaged, making them more susceptible to further injury during and after surgery.

Triggering receptors expressed on myeloid cells 2 (TREM2) are a membrane protein. TREM2 forms a receptor signaling complex with the TYRO protein tyrosine kinase-binding protein and causes Ca^2+^ mobilization in macrophages and the activation of serine/threonine protein kinase and extracellular signal-regulated kinase (ERK) [38]. TREM2 is highly expressed in the brain, lung, and adrenal glands, with a function related to extracellular waste materials’ elimination.

Soluble TREM2 (sTREM2) is produced by the proteolytic cleavage of the extracellular domain of TREM2 and can be passed through the brain–cerebrospinal fluid (CSF) barrier. Wang et al. (2022) reported on the postoperative upregulation of serum sTREM2 among open-heart surgery patients [15]. Interestingly, serum sTREM2 was found to be associated with mild cognitive impairment among obstructive sleep apnea patients, highlighting the effect of a reduced oxygen level with serum sTREM2 upregulation [39]. Thus, serum sTREM2 can be a good biomarker to predict possible cognitive impairment due to oxygen deprivation during open-heart surgery. Furthermore, elevated preoperative serum sTREM2 levels will ultimately increase the risk of developing POCD after open-heart surgery.

Reticulon 1 (RTN1) is highly expressed in the endoplasmic reticulum of the brain and is involved in the ER stress response. Elevated levels of RTN1 in the blood during the preoperative period can indicate several underlying mechanisms that contribute to cognitive decline post-surgery. Elevated levels of RTN1 might indicate heightened ER stress, which can lead to neuronal dysfunction, apoptosis, and neurodegenerative processes [40]. Elevated preoperative RTN1 levels could reflect ongoing neurodegenerative changes and underlying neuronal injury, making the brain more vulnerable to additional insults from surgery.

S100 calcium-binding protein B (S100B) is a calcium-binding protein predominantly localized in the cytoplasm or nucleus of astrocytes, a type of glial cell in the central nervous system. Increased S100B levels reflect astrocyte activation and an increased permeability of the BBB [41], making the brain more susceptible to inflammatory mediators and potential neurotoxins during and after surgery.

Silva et al. (2016) identified relationships between S100B protein levels and the timing of cognitive assessments following open-heart surgery. They suggest that S100B serum levels at the end of surgery can significantly predict POCD 21 days post-surgery, while S100B levels measured at 6 and 24 h after surgery can significantly predict POCD 180 days later [20]. These findings indicate the potential of S100B as a biomarker for the early identification of patients at risk of cognitive decline.

From the PPI analysis, the PPI network retrieved from the four DEPs reveals the involvement of gene ontology contributing to low-density lipoprotein particle binding (GO:0030169) and a disease–gene association related to cardiac arrest in POCD development (DOID:0060319). In agreement, an in vivo study found that hyperlipidemia in rats increased postoperative cognitive impairment following surgery [42]. In clinical studies, lowering the cholesterol level via medication is beneficial for neurological disorders and contributes to a significant reduction in POCD among postoperative patients [43].

The limitation of this systematic review is the low number of proteins gathered due to the low number of articles reporting on the preoperative protein expression, thus hindering the accurate integrated bioinformatic result. Furthermore, there was bias in the protein selection as all the studies did a targeted protein quantification according to their hypothesis and preferences. A whole-genome protein analysis or whole-genome protein sequencing would allow fewer protein selection bias data and provide a more reliable functional enrichment network and gene ontology for preoperative POCD among patients undergoing open-heart surgery.

## 4. Materials and Methods

This review has been officially registered in PROSPERO (No. CRD42024596225).

### 4.1. Search Strategy

The PRISMA guideline was used to conduct the systematic review. A thorough literature search was completed using PubMed, EBSCOhost, Science Direct, and Scopus databases. Relevant research articles released until 4 April 2024 were identified. MeSH terms from the Cochrane Library like postoperative cognitive complications, thoracic surgery, and protein were used to generate synonyms for keywords. Further keywords were discovered through the screening of related research articles. This combination of keywords was used as part of the search strategy: (“Postoperative cognitive decline” OR “Postoperative cognitive disorders” OR “Postoperative cognitive dysfunction” OR “Postoperative cognitive complications”) AND (“Thoracic Surgery” OR “Cardiac Surgery” OR “Heart Surgery”) AND (“Protein expression” OR proteomic OR “Protein profiling”). The bibliographies of the retrieved papers were used to find further references.

### 4.2. Inclusion Criteria

Studies that engaged cardiac patients aged 18 and above undergoing cardiac surgery were selected in this systematic review. Studies that took peripheral blood before and after surgery as their sample for protein analysis were selected. Only studies that provide protein expression data were included in this study.

### 4.3. Exclusion Criteria

Editorials, case reports, conference proceedings, and narrative review articles were excluded because they had no primary data. In silico, in vitro, in vivo, and animal models were also excluded. Intervention trials involving the use of oximetry were excluded from consideration.

### 4.4. Screening of Articles for Eligibility

**Figure 2:**
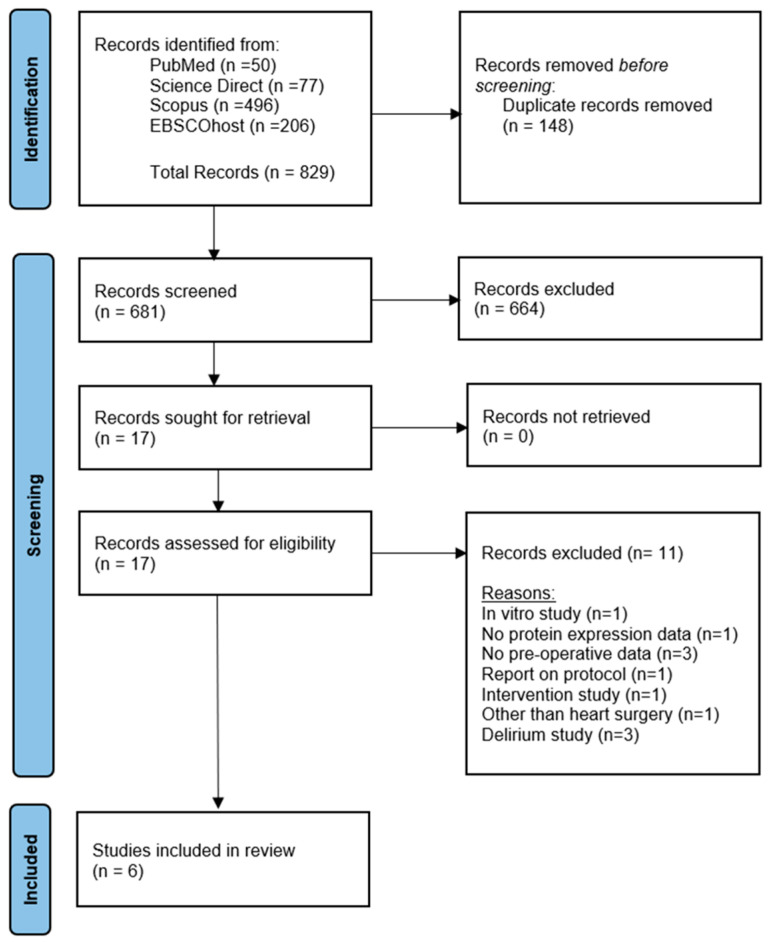
PRISMA flow diagram was used for study selection in this systematic review.

Three screening stages were carried out to select the most relevant articles gathered from all resources. Duplicates were eliminated and all articles with irrelevant titles were excluded from the first screening. The abstracts of the remaining papers were reviewed, and those that did not fit the inclusion criteria were eliminated in the second screening. Lastly, the full text of the remaining articles was thoroughly examined. Every article that did not fit the requirements for inclusion was eliminated in this third screening. All authors participated in the screening, choosing, and extracting data exercises. Whenever conflicts appeared, they were discussed, and the majority decided. Reasons for rejection were noted in the records. The PRISMA flow diagram in Figure 2 summarizes the article assortment procedure and the reason for article removal.

### 4.5. Data Extraction

Data were extracted from all the selected studies that met the inclusion requirements. The extracting processes of the data involve all the authors. A data collection form was used to standardize the data-gathering process. The information collected is as follows: (1) author name; (2) year of publication; (3) article title; (4) study design; (5) sample size; (6) sample type; (7) type of anesthesia; (8) methods used to determine POCD; (9) protein expression method; (10) protein expression result; and (11) conclusion. Table 1 summarizes the details of the collected data.

### 4.6. Study Quality Assessment

The study quality of all the included studies was accessed independently by MHE, NAH, NJN, and SMM, using the Joanna Briggs Institute critical assessment tools [44,45]. SA, SK, and KMH verified the quality of the studies. Studies with an overall score of less than 50% were classified as low-quality studies (high risk of bias). Studies with an overall score between 50% and 69% were classified as moderate-quality studies (moderate risk of bias) and studies with a score of more than 69% were classified as high-quality studies (low risk of bias).

### 4.7. Protein–Protein Interaction (PPI) Network, Clustering, and Visualization

All significant differentially expressed proteins (DEPs) from all studies were pooled and analyzed for PPI network and functional enrichment analysis by utilizing STRING software (version 12.0; https://string-db.org/) (Lausanne, Switzerland) [46]. The analysis resulting from STRING was exported into Cytoscape software (version 3.8.0; http://www.cytoscape.org/) (Seattle, WA, USA) to visualize its molecular interaction networks and integrate the protein expression profiles of the DEPs [47]. The module analysis of the target network and protein clustering were completed using Cytoscape MCODE plug-in. A degree cut-off = 2, node score cut-off = 0.2, node density cut-off = 0.1, K-score = 2, and max depth = 100 were used as the module-selection criteria.

### 4.8. Gene Ontology (GO) and Pathway Enrichment Analysis

Database for Annotation, Visualization, and Integrated Discovery (DAVID) were used to analyze all genes in each cluster. Gene ontology that exhibited significant functional-annotation enrichment related to POCD was identified from the analysis using DAVID [48,49]. The genes’ involvement in the pathways related to POCD was identified based on the Kyoto Encyclopedia of Genes and Genomes (KEGG) pathway.

## 5. Conclusions

Identifying a group of candidate proteins for the preoperative prediction of POCD could significantly improve patient management and outcomes. However, further studies are warranted to identify biomarkers with a high predictive value for POCD among patients undergoing open-heart surgery. Thus, for further studies, circulating biomarkers related to chronic mild hypoxia, the blood–brain barrier, neuroinflammation, depression, and nutrition are proposed to be used as the serum preoperative POCD biomarkers.

## Figures and Tables

**Table 1:** Characteristics summary of the included studies.

Author, Year (References)	Title	Country	Study Design	Sample Size	Anesthesia	POCD Determination Method	Protein Expression Method	Types of Open-Heart Surgery
Wang et al., 2022 [15]	Anesthesia- and surgery-induced elevation of CSF sTREM2 is associated with early cognitive dysfunction after thoracoabdominal aortic dissection surgery	China	cross sectional	POCD = 34, nonPOCD = 48	propofol, sevoflurane, sufentanil, and cisatracurium	Mini Mental State Examination (MMSE), symbol digit modalities test (SDMT), digit span forward test (DSFT), and digit span backward test (DSBT), Rey auditory verbal learning test (RAVLT), Stroop color–word test (SCWT), and the confusion assessment method-intensive care unit (CAM-ICU)	enzyme-linked immunosorbent assay (ELISA)	Thoracoabdominal aortic replacement
Nurcahyo et al., 2021 [16]	An Association Between C-Reactive Protein Levels and the Occurrence of Cognitive Dysfunction After Heart Valve Replacement	Indonesia	cross sectional	POCD = 28, nonPOCD = 4	Midazolam, Sevoflurane, fentanyl, rocuronium	Indonesian version of Montreal Cognitive Assessment (MoCA-INA)	immunoturbidimetric using Cobas c501 autoanalyzer	Heart valve replacement
Szwed et al., 2020 [17]	Novel Markers for Predicting Type 2 Neurologic Complications of Coronary Artery Bypass Grafting	Poland	case-control study	POCD = 13, nonPOCD = 13	Fentanyl, etomidate, propofol, sevoflurane	Stroop test, Trail Making Test, Digit Span Test, and Rey Auditory Verbal Learning Test	ELISA	Elective isolated off-pump CABG
Baktiar et al., 2020 [18]	S100B as a serologic marker for cognitive dysfunction following open-heart surgery	Indonesia	cross sectional	POCD = 31, nonPOCD = 24	N/A	Immediate and delayed Rey auditory verbal learning test, the trail making test A and B, and the digit span forward and backward test	ELISA	Not specified
He at al., 2017 [19]	The significance of S100β protein on postoperative cognitive dysfunction in patients who underwent single valve replacement surgery under general anesthesia	China	case-control study	POCD = 32, nonPOCD = 146	Midazolam, etomidate, fentanyl, and cisatracurium	Mini Mental State Examination (MMSE) and Montreal Cognitive Assessment (MoCA)	ELISA	Single valve replacement
Silva et al., 2016 [20]	S100B protein and neuron-specific enolase as predictors of cognitive dysfunction after coronary artery bypass graft surgery	Brazil	cross sectional	POCD = 23, nonPOCD = 65	Fentanyl or sufentanil, etomidate, propofol or midazolam, pancuronium bromide, or cisatracurium besylate	Telephone Interview Cognitive Status (TICS), Verbal Learning Test (VLT), Stroop Color–Word Test (SCWT), Trail Making Test (TMT), and Symbol Digit Modalities Test (SDMT)	ELISA	CABG

**Table 2:** Statistical significance of differences in the baseline characteristics between POCD and nonPOCD groups.

	POCD							NonPOCD						
References	Age (Year)	Gender (Male%)	BMI (kg/m^2^)	Education (Year)	Comorbidities	Smoking (%)	Drinking (%)	Age (Year)	Gender (Male%)	BMI (kg/m^2^)	Education (Year)	Comorbidities	Smoking (%)	Drinking (%)
Wang et al., 2022 [15]	60.2 ± 6.8 *	70.6	25.4 ± 3.2	8 ± 3 *	NS	50	41.2	44.1 ± 8.1 *	77.1	25.6 ± 3.3	9 ± 2	NS	31.8	27.1
Nurcahyo et al., 2021 [16]	NA	NA	NA	NA	NA	NA	NA	NA	NA	NA	NA	NA	NA	NA
Szwed et al., 2020 [17]	65.7 ± 7.0	66.7	29.1 ± 4.6	12	NS	53.8	NA	64.0 ± 5.0	73.1	27.6 ± 5.0	12	NS	33.3	NA
Baktiar et al., 2020 [18]	55.4 ± 11.7	64.5	24.2 ± 4.0	NA	NS	NA	NA	50.9 ± 13.6	66.7	25.4 ± 4.3	NA	NS	NA	NA
He at al., 2017 [19]	54.5 ± 12.7	NA	21.7 ± 1.2	NA	NA	NA	NA	55.6 ± 10.8	NA	20.7 ± 1.3	NA	NA	NA	NA
Silva et al., 2016 [20]	NA	NA	NA	NA	NA	NA	NA	NA	NA	NA	NA	NA	NA	NA

Comorbidities included diabetes mellitus, hypertension, hyperlipidemia, and renal impairment. * indicates a significant difference (*p* < 0.05). NA: Not available (the data were not reported in the published paper). NS: Not significant (*p* > 0.05).

**Table 3:** Reported differentially expressed proteins in the patients identified as having POCD.

Author, Year (References)	Preoperative			Postoperative		
	Upregulated	Downregulated	No Significant Differences	Upregulated	Downregulated	No Significant Differences
Wang et al., 2022 [15]	-	-	TREM2, Aβ42, P-tau, T-tau	TREM2	-	Aβ42, P-tau, T-tau
Nurcahyo et al., 2021 [16]	CRP	-	-	CRP	-	-
Szwed et al., 2020 [17]	-	-	GFAP, NSP, pNfH, and VILIP-1	RTN1	-	GFAP, pNfH, and VILIP-1
Baktiar et al., 2020 [18]	-	-	S100B	-	-	S100B
He at al., 2017 [19]	-	-	S100B	S100B	-	-
Silva et al., 2016 [20]	-	-	S100B, NSE	S100B	-	NSE

## Data Availability

No new data were created or analyzed in this study. Data sharing is not applicable to this article.

## Supplementary Materials

The following supporting information can be downloaded at: https://www.mdpi.com/article/10.3390/ijms252212238/s1.
