# Supplementary material for: Preoperative Protein Profiling Among Postoperative Cognitive Dysfunction (POCD) Patients Following Open-Heart Surgery: A Systematic Review and Integrated Bioinformatic Analysis
Source: Int J Mol Sci. 2024 Nov 14;25(22):12238. doi: 10.3390/ijms252212238 (PMC11595097; doi:10.3390/ijms252212238)
Supplement: Supplementary file 1 [file ijms-25-12238-s001.zip › ijms-3242181-supplementary.pdf]

Table S1 Study quality assessment using Joanna Briggs Institute critical assessment tools

a) Case-control studies assessment

Study	Comparable groups	Appropriate matching	Same criteria for identification	Standard exposure measurement	Same exposure measurement	Confounding factors identified	Strategies for confounding	Standard outcome assessment	Meaningful exposure period	Appropriate statistical analysis	Overall quality
Szwed et al., 2020 (18)	Yes	Yes	Yes	Yes	Yes	Yes	Yes	Yes	Yes	Yes	High
He et al., 2017 (20)	Yes	Yes	Yes	Yes	Yes	Yes	No	Yes	Yes	Yes	High

b) Cross-sectional studies assessment

Study	Clear inclusion criteria	Detailed description of subjects and setting	Valid and reliable exposure measurement	Objective, standard criteria for condition	Confounding factors identified	Strategies for confounding	Valid and reliable outcome measurement	Appropriate statistical analysis	Overall quality
Wang et al., 2022 (16)	Yes	Yes	Yes	Yes	Yes	Yes	Yes	Yes	High
Nurcahyo et al., 2021 (17)	Yes	Yes	Yes	Yes	Yes	No	Yes	Yes	High
Baktiar et al., 2020 (19)	Yes	Yes	Yes	Yes	No	No	Unclear	Unclear	Moderate
Silva et al., 2016 (21)	Yes	Yes	Yes	Yes	No	No	Yes	Yes	High
